# Habitat use affects morphological diversification in dragon lizards

**DOI:** 10.1111/j.1420-9101.2010.01971.x

**Published:** 2010-05

**Authors:** D C COLLAR, J A SCHULTE, B C O’MEARA, J B LOSOS

**Affiliations:** * Department of Organismic and Evolutionary Biology and Museum of Comparative Zoology, Harvard UniversityCambridge, MA, USA; † Department of Biology, Clarkson UniversityPotsdam, NY, USA; ‡ Department of Ecology and Evolutionary Biology, University of TennesseeKnoxville, TN, USA

**Keywords:** Agamidae, Brownian motion, ecomorphology, Iguania, locomotion, phylogenetic comparative method

## Abstract

Habitat use may lead to variation in diversity among evolutionary lineages because habitats differ in the variety of ways they allow for species to make a living. Here, we show that structural habitats contribute to differential diversification of limb and body form in dragon lizards (Agamidae). Based on phylogenetic analysis and ancestral state reconstructions for 90 species, we find that multiple lineages have independently adopted each of four habitat use types: rock-dwelling, terrestriality, semi-arboreality and arboreality. Given these reconstructions, we fit models of evolution to species’ morphological trait values and find that rock-dwelling and arboreality limit diversification relative to terrestriality and semi-arboreality. Models preferred by Akaike information criterion infer slower rates of size and shape evolution in lineages inferred to occupy rocks and trees, and model-averaged rate estimates are slowest for these habitat types. These results suggest that ground-dwelling facilitates ecomorphological differentiation and that use of trees or rocks impedes diversification.

## Introduction

One of the great questions in evolutionary biology concerns the causes of differences in diversity among clades. Ecological factors are often implicated to explain this pattern because the ecological circumstances available to the members of a lineage contribute to the mode of natural selection they experience and thus shape ecological divergence, morphological adaptation and the evolution of new species. Although much work has focused on the role of biotic interactions within communities (e.g. competition-driven divergent selection in *Anolis* lizards [Williams, 1972; Losos, 2009], Hawaiian silverswords [Carlquist *et al.*, 2003] and Darwin’s finches [Schluter, 1988; Grant & Grant, 2008]), other aspects of a lineage’s ecology may also be important for diversification. In this study, we test the hypothesis that diversity varies as a function of habitat.

For many reasons, some habitats may foster greater diversity than others. Some habitat types may be readily subdivided, perhaps because of spatial complexity (e.g. coral reefs [Alfaro *et al.*, 2007]) or geographical area (e.g. arid habitats in Australia [Rabosky *et al.*, 2007]) and may thereby present evolutionary lineages with many alternative means for microhabitat specialization or local adaptation. Other habitats may impose stringent functional constraints that lead to strong selection resisting ecological and phenotypic divergence away from an adaptive peak (Butler & King, 2004; Collar *et al.*, 2009). Habitat types may also contribute differently to diversification because they vary in the number and type of species interactions they present, such as the presence or absence of predators (McPeek & Brown, 2000). In addition, some habitats may provide opportunities if they are variable across space in the strength of species interactions (McPeek, 1996) or in their functional demands.

The consequences of habitat use for diversification have been investigated primarily in the context of explaining variation in species richness among clades. Evolutionary transitions between habitat types that differ in the opportunities they provide for ecological divergence are often implicated to explain shifts in rates of lineage diversification. For example, several invasions of coral reefs in tetraodontiform fish lineages are temporally coincident with increases in rates of diversification (Alfaro *et al.*, 2007), the transition into arid habitats is associated with elevated rates of diversification in Australian skinks (Rabosky *et al.*, 2007), and diversification rates in damselflies vary across gradients of pond permanence (McPeek & Brown, 2000).

One explanation for these associations between habitat use and species richness is that the process of lineage splitting is mechanistically linked to niche differentiation. However, species richness and ecological diversity need not be correlated during evolution (Foote, 1993; Losos & Miles, 2002; Adams *et al.*, 2009), and elevated rates of lineage diversification within a habitat type do not require increases in rates of ecological evolution. Indeed, the neutral theory of biodiversity emphasizes the extent to which species diversification may occur in the absence of ecological differentiation (Hubbell, 2001).

Using morphological variation in ecologically relevant characters as a surrogate for ecological variation, we asked whether a relationship exists between habitat use and ecological diversity. We focused on dragon lizards (Agamidae), an ecologically and morphologically diverse radiation of iguanian lizards comprising roughly 400 species distributed throughout the Old World. Agamid lizards vary in their structural habitat use, including species that primarily use rocks, trees or terrestrial surfaces as well as some semi-arboreal species that frequently use both trees and terrestrial surfaces. Because the ability to move about and hold position in the environment is partly a consequence of structural habitat use and because movement is important to the performance of ecological tasks, such as foraging, evading predation and defending territory (Losos, 1990; Irschick & Garland, 2001), these four types of habitat use may contribute differently to ecomorphological diversification in agamid lineages.

To evaluate this hypothesis, we applied a phylogenetic approach that tests for associations between habitat and rates of morphological evolution in agamid lineages. We inferred phylogenetic relationships for 90 agamid species based on mitochondrial DNA sequences, reconstructed ancestral habitat use and used these reconstructions as the basis for fitting models of evolution to species values for morphological traits. We then compared fit and parameter estimates for models that differ in the number of evolutionary rates (based on the Brownian motion model), where rates are allowed to vary among lineages inferred to use different habitat types. We interpreted habitat types associated with high rates of morphological evolution to be those that facilitate ecological divergence.

## Materials and methods

### Reconstructing phylogeny

We reconstructed phylogenetic relationships for 90 agamid species—representing nearly one-quarter of the group’s recognized species diversity—as well as four outgroup species. Our molecular data set included 1.2 kb of mitochondrial DNA including partial sequences for the protein-coding genes, NADH dehydrogenase subunit 1 (ND1) and cytochrome c oxidase subunit I (COI), and the complete sequence for NADH dehydrogenase subunit 2 (ND2). This analysis excluded intervening tRNA-coding regions because they are highly variable among the sampled taxa, making unambiguous alignment of these regions difficult and potentially unreliable (Schulte *et al.*, 2004a; Schulte & de Queiroz, 2008). All sequences were obtained from GenBank (accession numbers are in Table S1) and aligned by eye. Base positions inferred to have ambiguous homology at the ends of ND1 and ND2 were excluded from phylogenetic analyses (198 of 1281 aligned positions). Alignment is available in TreeBASE (Study accession number S2669, Matrix accession numbers M5148; to be added upon acceptance of manuscript).

We used these sequences to simultaneously infer phylogenetic relationships among agamid species and estimate branch lengths in relative time using Bayesian phylogenetic analysis and a relaxed molecular clock approach implemented in the program beast (Drummond *et al.*, 2006; Drummond & Rambaut, 2007). We partitioned mtDNA sequences by codon position and, for each partition, separately fit a general time reversible model of nucleotide substitution that allows for gamma-distributed substitution rate variation among sites and invariant sites (Yang, 1994) because previous analysis of these sequences for a subset of the agamid species included in this study showed that this model provided the best fit relative to simpler substitution models (Schulte *et al.*, 2004a). Variation in substitution rates among lineages was modelled by a lognormal distribution in which the mean rate was set to 1.0 (i.e. no external calibration was used to estimate divergence times), and no correlation was assumed between ancestor and descendant branches (Drummond *et al.*, 2006; Drummond & Rambaut, 2007). Uninformative priors were applied for all parameter estimates.

We used beast to sample the posterior probability distribution of phylogenetic trees and substitution model parameters given species’ sequence data according to a Markov chain Monte Carlo (MCMC) algorithm (Drummond & Rambaut, 2007), which we ran twice for 25 × 10^6^ generations per run. For each run, a random starting tree was generated under a Yule (pure-birth) process (Drummond & Rambaut, 2007), and the first 2.5 × 10^6^ generations were discarded as burn-in. We verified the adequacy of sampling from the posterior probability distribution using the program tracer (Drummond & Rambaut, 2007) to determine that effective sample sizes for model parameter estimates were greater than 200 (Drummond *et al.*, 2006) and confirmed convergence of the two MCMC runs using the program awty (Nylander *et al.*, 2008) by inspecting the correlation between split frequencies.

The central question of this study focuses on the role of structural habitat use in morphological evolution. Although reconstruction of agamid phylogeny is necessary to address this question, strong inference about a single, best phylogeny is not. Rather than base our analyses on a single consensus tree, we retained a set of 1000 phylogenies sampled from the posterior distribution for use in ancestral state reconstructions (see next section). To do this, we randomly sub-sampled 500 trees from each of the MCMC runs. Because MCMC algorithms of beast sample trees in proportion to their posterior probability, performing subsequent analyses on this set of trees incorporates uncertainty in agamid phylogeny into our analyses in a manner similar to the method of Huelsenbeck & Rannala (2003).

### Reconstructing ancestral habitat use

To reconstruct ancestral habitat use in agamid lineages, we used stochastic character mapping, which is a Bayesian method that implements MCMC to sample character reconstructions in proportion to their posterior probability under a Markov process of state transitions given species’ character states and a phylogeny (Nielsen, 2002; Huelsenbeck *et al.*, 2003; Bollback, 2006). We assigned structural habitat types—rock-dwelling, arboreal, terrestrial and semi-arboreal—to the 90 agamid species included in our phylogenetic analysis based on Stuart-Fox & Owens’s (2003, their online appendix 3) and Stuart-Fox & Ord’s (2004, their online appendix A) syntheses of ecological data in Agamidae. Our data set excluded species that these studies categorized as generalists—those that are reported to occur on rocks, trees and terrestrial surfaces—because this category appeared to contain a heterogeneous set of species, including species comprised of generalized individuals, polymorphic populations or populations that vary in habitat use. We used the program simmap (Bollback, 2006) to generate 10 stochastic maps of habitat use for each of the 1000 phylogenies sampled from our phylogenetic analysis (described earlier). From the resulting 10 000 habitat use reconstructions, we randomly sampled 500 for use in model-fitting analyses. The set of 500 reconstructions thus represents sampling from the posterior probability distributions of both trees and ancestral reconstructions. Because the MCMC algorithms of beast (Drummond & Rambaut, 2007) and simmap (Bollback, 2006) sample trees and character state histories, respectively, in proportion to their posterior probabilities, use of this set of structural habitat reconstructions in subsequent model-fitting analyses allowed us to integrate over uncertainty in both phylogeny and ancestral states.

### Quantifying species’ morphology

We quantified species values for morphological features of the body and limbs that have functional consequences for iguanian lizard locomotor performance (e.g. Reilly & Delancey, 1997; Irschick & Jayne, 1999; Jayne & Irschick, 1999; Spezzano & Jayne, 2004), including snout-vent length, tail length, body width, pectoral width, pelvic width, humerus length, ulna length, carpal length, IV metacarpal length, femur length, tibia length, tarsal length and IV metatarsal length. Descriptions of landmarks used to delimit these morphological variables are detailed in Schulte *et al.* (2004b). Species values are means of measurements made on preserved specimens of adult males and females (species data are available from the authors by request). Sample sizes within species ranged between one and 36 individuals (median = four individuals; Table S1).

To account for correlations between variables and to reduce the dimensionality of the morphological data set, we performed principal components analysis (PCA) on the correlation matrix of agamid species values. Species scores on principal component (PC) axes were then used as species character values in subsequent model-fitting analysis. Additionally, we quantified sampling error for species PC scores. We used the eigenvalues and eigenvectors from PCA on species values to transform morphological values for all individuals into PC scores and estimated the pooled within-species variance for each PC. Each species’ sampling variance was then taken as the pooled within-species variance divided by the number of individuals sampled for that species.

### Fitting models of morphological evolution

To assess the effects of structural habitats on morphological diversification in Agamidae, we fit several models of evolution to species’ PC scores and reconstructions of agamid phylogeny and ancestral habitat states. We examined Brownian motion models of character evolution that differed in the number of rates of evolution, defined as the time-independent variance parameter, σ^2^, of the Brownian motion model of character evolution (Felsenstein, 1985, 1988; Garland, 1992; Martins, 1994; Collar *et al.*, 2005; O’Meara *et al.*, 2006; Thomas *et al.*, 2006). These models specified separate evolutionary rates for lineages inferred to use different habitat types following O’Meara *et al.* (2006) and Collar *et al.* (2009). Inferred habitat states in agamid lineages were based on ancestral reconstructions from simmap (Bollback, 2006). The most complex model specifies separate rates for rock-dwelling, arboreal, semi-arboreal and terrestrial lineages (4-rate full: 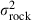, 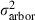, 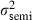, 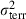), whereas the simplest model specifies a single rate for all agamid lineages, regardless of inferred habitat state (1-rate no effect: 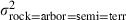).

We also explored the fit of eight additional models in which the effects of some habitat types were assumed to be equal. We note that the following models are not an exhaustive set of all possible models given these four habitat categories, but rather a subset of models that we deemed most plausible based on hypothesized shared and unique properties of the different surface types. We fit three three-rate models that set rate categories to be equivalent according to possible shared effects of habitats: the effects of arboreality and semi-arboreality may be the same because these habitat types both require that species occur in forests and move along and cling to trees, which may result in similar ecological and functional demands (3-rate shared tree effect: 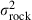, 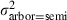, 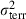); alternatively, the effects of terrestrial and semi-arboreal habitats may be equivalent if the demands of ground surfaces prevail over those of trees in semi-arboreal species (3-rate shared ground effect: 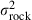, 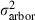, 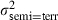); additionally, because both rocks and trees present steeply inclined surfaces to the species that use them, rock-dwelling and arboreality may have similar effects in agamid lineages (3-rate shared incline effect: 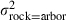, 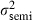,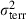). We also fit four two-rate models to test for unique effects of each habitat type: the unique effect of rock-dwelling (2-rate rock effect: 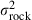, 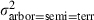); of arboreality (2-rate arboreal effect: 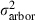, 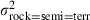); of semi-arboreality (2-rate semi-arboreal effect: 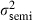, 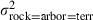) and of terrestriality (2-rate terrestrial effect: 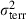, 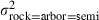). Finally, because species that predominantly use steeply inclined surfaces (rock-dwelling and arboreal species) may experience demands that differ from species that use the ground frequently (terrestrial and semi-arboreal), we fit a fifth two-rate model that separates the effects of steeply inclined surfaces from those of ground-dwelling (2-rate incline-ground: 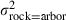, 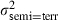).

To evaluate the effects of different habitat states on morphological evolution, we fit multiple-rate Brownian motion models rather than multiple-peak Ornstein–Uhlenbeck (OU) models, which describe evolution under selection towards fixed phenotypic optima (Felsenstein, 1988; Hansen & Martins, 1996; Hansen, 1997; Butler & King, 2004). This decision was based on two considerations. First, multiple-rate Brownian models allowed us to assess whether the process of diversification differed across lineages characterized by different evolutionary regimes (O’Meara *et al.*, 2006; Thomas *et al.*, 2006; Collar *et al.*, 2009). In contrast, current multiple-peak OU models allow inferences to be made about the positions of phenotypic optima corresponding to different selective regimes, but they assume that the process of evolving towards those values (i.e. the strength of selection and the magnitude of the rate of stochastic change) is the same for all selective regimes (Hansen, 1997; Butler & King, 2004; Scales *et al.*, 2009). Because our goal was to test the hypothesis that habitat types contribute differently to the process of evolution, we were more interested in evaluating habitat-associated variation in the rate of morphological change than in finding fixed adaptive morphologies. Second, Brownian motion has been shown to adequately describe adaptive evolution under a variety of evolutionary conditions (e.g. when environmental change causes shifts in the position of adaptive peaks, or when unconsidered lineage-specific differences in selection, environment or genetics have large effect relative to the strength of selection because of the considered selective regime [Hansen & Martins, 1996; Hansen, 1997]). Nevertheless, we note that both Brownian motion and OU processes are relatively simple models used to describe complex reality, and the multiple-rate models we chose to fit were those that we deemed most appropriate for evaluating our hypothesis given the aforementioned considerations.

We used the computer program Brownie 2.1 (O’Meara *et al.*, 2006; O’Meara, 2008) to fit each of the 10 models to species scores for each PC across the 500 habitat reconstructions. The method uses maximum likelihood and extends the noncensored approach of O’Meara *et al.* (2006) to accommodate models that specify multiple evolutionary rates for phylogenetic branches associated with states of a categorical variable (in this case, habitat use). The method also incorporates sampling error for species values using the approach of Martins & Hansen (1997). For each of the four PCs, we fit each model to the set of species’ scores iterating over the 500 habitat reconstructions. This process resulted in distributions of model parameter estimates and fit scores for each combination of model and PC, and the spread of the distributions represent variation that is because of uncertainty in phylogeny and ancestral habitat reconstructions.

To assess model fit, we used the Akaike information criterion (AIC), which is a function of the likelihood, *L*, of the data given the model and the number of parameters, *k*: AIC = 2*k* − 2ln (*L*). More specifically, we used AICc, which is AIC with a correction for small sample size—appropriate when the number of observations, in this case species, is < 40 times the number of estimated parameters (Burnham & Anderson, 2002). Lower AICc scores indicate better fit. To select the best fitting model for each PC given uncertainty in phylogeny and ancestral habitats, we evaluated the average model fit over the 500 reconstructions and compared mean AICc across models. We note that averaging AICc in this way is valid because the data for species are the same for all iterations of model fitting. The 500 reconstructions should not be considered as different, independent data sets but as alternative estimates of phylogeny and ancestral habitat states sampled in proportion to their posterior probabilities given the same data, and averaging AICc over this sample allowed us to quantify model fit in a way that integrates over uncertainty in these estimates. We also calculated Akaike weights from mean AICc as an additional descriptor of each model’s fit to each PC. Akaike weights describe the proportion of support a model receives relative to the total support available for all models (Burnham & Anderson, 2002).

In addition to comparing mean AICc, we also explored the sensitivity of model selection to alternative phylogeny and habitat reconstructions. We compared AICc among models on each reconstruction and generated distributions for ΔAICc—the difference between each model’s AICc and the best fitting model’s AICc.

We were unable to unambiguously select a single best fitting model for any of the PCs because several models received substantial support; ΔAICc was < 2 (Burnham & Anderson, 2002) and several models provided the best fit for a similar proportion of the reconstructions. To assess the effect of habitat on morphological evolution in light of this uncertainty in model selection, we compared model-averaged estimates of the rate of evolution associated with each habitat use type, where the model-averaged rate is the average rate across all models weighted by each model’s Akaike weight (Burnham & Anderson, 2002). This weighting strategy ensures that parameter estimates from better supported models count more towards the overall model-averaged rate estimates. The resulting model-averaged rates of PC evolution for each habitat state thus average across uncertainty in the model of character evolution as well as uncertainty in the reconstruction of agamid phylogeny and ancestral habitat states.

## Results

**Fig. 1:**
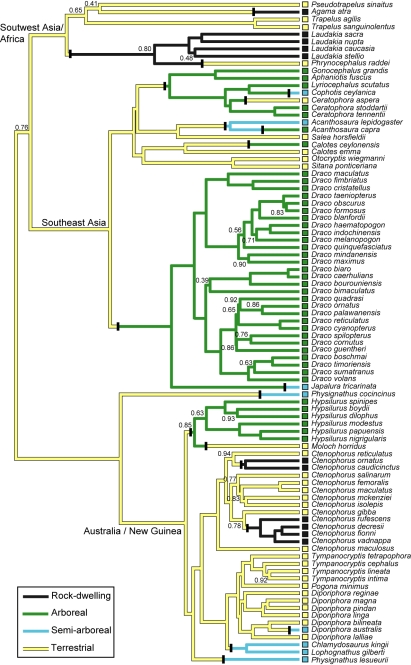
Maximum clade credibility phylogeny for 90 agamid species illustrating a single reconstruction of structural habitat use. Nodes are supported by > 0.95 Bayesian posterior probabilities unless otherwise noted, and branch lengths are proportional to time (i.e. root node depth is 1.0). Colour/shade of branches indicates inferred habitat use based on stochastic character mapping (see key). Habitat states for species are given by colour/shade of terminal nodes. Transitions between habitat states are highlighted by vertical, black bars. We used this reconstruction of phylogeny and habitat state and 499 others as the basis for fitting models of evolution in which rates of morphological diversification are allowed to differ in lineages that use different habitat types.

Bayesian phylogenetic analysis yielded a set of 1000 ultrametric trees that are generally consistent with previous phylogenetic hypotheses involving agamid lizards (Macey *et al.*, 2000; McGuire & Kiew, 2001; Melville *et al.*, 2001; Schulte *et al.*, 2004a; Hugall *et al.*, 2008). Figure 1 shows the topology and branch lengths in relative time for the maximum clade credibility tree (i.e., the tree with the greatest posterior probability summed over all nodes) from our sample of the posterior distribution; we summarize node support on this tree by identifying nodes whose posterior probabilities are less than 0.95. We found strong support for the monophyly of three recognized biogeographical groups, a clade of southwest Asian and African species, a clade of southeast Asian agamids, and a clade of species from Australia and New Guinea which is the sister group to the southeast Asian species, *Physignathus concincinus*.

Stochastic mapping of habitat use across the set of agamid phylogenies provides strong support for multiple, independent transitions to each of the four structural habitat types. All sampled reconstructions infer multiple origins of semi-arboreality (median = 7, minimum = 5; maximum = 10), more than 99% of reconstructions infer multiple origins of rock-dwelling (median = 4, maximum = 7 origins) and terrestriality (median = 5, maximum = 10 origins), and 91.3% reconstruct more than one origin of arboreality (median = 3, maximum = 7 origins). Figure 1 depicts one stochastic habitat reconstruction from simmap (Bollback, 2006) on the maximum clade credibility tree and shows the median number of origins of each habitat type.

**Fig. 2:**
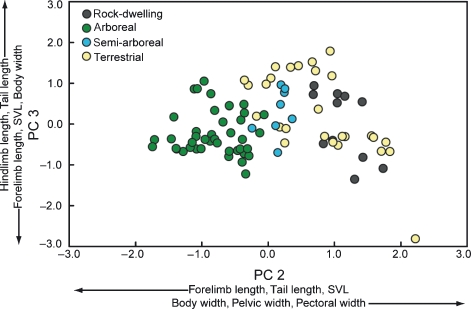
Scatterplots of agamid species in a morphospace defined by principal components 2 and 3. Color-coding for species’ habitat states is the same as in Fig. 1. Loadings of original variables on PCs are described for each axis. For brevity, we use ‘hindlimb length’ and ‘forelimb length’ to describe loadings on PCs when more than one element of that limb loads strongly on that axis. See Table 2 for details about PCA.

PCA on species values for morphological traits provides four axes that together account for 96.3% of the total variation between species. PC 1 accounts for 82.9% of the variation and loads strongly and similarly across all variables (Table 1); we interpreted PC 1 to represent variation among species that is because of differences in size. The three subsequent PCs collectively explain 78.0% of the variation in shape (i.e., the variation not explained by PC 1). Loadings for PCs on the original morphological variables are reported in Table 1 and the distribution of species on PCs 2 and 3 is shown in Fig. 2. Notably, PC 2 separates arboreal species from species that use the other habitat types; all arboreal species have negative PC 2 scores, reflecting generally long, narrow bodies and relatively long forelimbs, whereas nearly all other species have positive scores on this axis, indicative of shorter, wider bodies and relatively short forelimbs (Fig. 2). Habitat groups do not appear to separate as clearly on the other shape axes (PC 3 and 4) or on the size axis (PC 1).

**Table 1:** Loadings of morphological variables on principal components (PC). Principal components analysis was performed on the correlation matrix of log-transformed agamid species trait values. Bold values indicate loadings considered strong (> |0.20|).

Character	PC 1	PC 2	PC 3	PC 4
Snout-vent length	**0.28**	**−0.21**	**−0.31**	0.17
Tail length	**0.26**	**−0.36**	**0.35**	**0.32**
Body width	**0.25**	**0.48**	**−0.21**	**−0.31**
Pectoral width	**0.28**	**0.33**	**−**0.09	**0.32**
Pelvic width	**0.26**	**0.38**	**−**0.08	**0.56**
Humerus length	**0.28**	**−0.23**	**−0.35**	**−**0.15
Ulna length	**0.28**	**−0.26**	**−0.28**	**−0.21**
Carpal length	**0.30**	0.08	**−**0.14	0.05
IV metacarpal length	**0.27**	**−0.38**	**−**0.04	0.14
Femur length	**0.29**	**−**0.04	**−**0.01	**−0.35**
Tibia length	**0.29**	0.13	**0.25**	**−0.34**
Tarsal length	**0.28**	0.20	**0.40**	**−**0.15
IV metatarsal length	**0.27**	**−**0.09	**0.53**	**−**0.01
Eigenvalue	10.78	0.89	0.61	0.24
% Total variation	82.93	6.81	4.68	1.83
% Shape variation	–	39.88	27.44	10.72

**Table 2:** Parameter estimates and model fitting for multiple-rate Brownian models in Agamidae, where evolutionary rates are associated with inferred habitat use states. Values are means ± standard errors from model fitting across 500 reconstructions of habitat use on agamid phylogenies sampled from posterior probability distribution. Bold denotes the best fitting model.

Character	Model	Root state	Rate rock	Rate terr	Rate semi	Rate arbor	−ln L	AICc	ΔAICc	Weight
PC 1	4-rate full 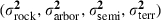	**1.457 ± 0.425**	**2.239 ± 2.062**	**20.334 ± 4.668**	**146.070 ± 46.015**	**13.221 ± 2.150**	**203.45 ± 1.81**	**417.61 ± 3.63**	**0.00**	**0.482**
	3-rate shared tree effect 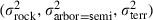	1.877 ± 0.389	2.036 ± 1.780	27.429 ± 5.480	18.238 ± 2.949	18.238 ± 2.949	209.54 ± 1.74	427.54 ± 3.47	9.93	0.003
	3-rate shared ground effect 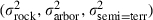	1.745 ± 0.462	1.959 ± 1.632	35.199 ± 4.287	35.199 ± 4.287	12.292 ± 1.993	206.60 ± 2.12	421.68 ± 4.25	4.07	0.063
	3-rate shared incline effect 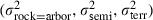	1.106 ± 0.286	11.586 ± 1.959	20.294 ± 4.645	148.404 ± 48.172	11.586 ± 1.959	205.54 ± 1.73	419.56 ± 3.47	1.94	0.183
	2-rate rock effect 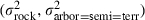	1.846 ± 0.340	2.198 ± 1.967	21.473 ± 1.233	21.473 ± 1.233	21.473 ± 1.233	210.08 ± 1.59	426.45 ± 3.19	8.83	0.006
	2-rate terrestrial effect 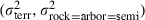	1.558 ± 0.290	15.370 ± 2.378	28.997 ± 4.786	15.370 ± 2.378	15.370 ± 2.378	212.55 ± 1.68	431.37 ± 3.36	13.76	0.000
	2-rate semi-arboreal effect 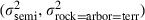	0.901 ± 0.159	14.125 ± 0.864	14.125 ± 0.864	166.730 ± 51.746	14.125 ± 0.864	206.38 ± 1.60	419.03 ± 3.20	1.42	0.238
	2-rate arboreal effect 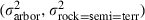	1.315 ± 0.262	27.538 ± 2.628	27.538 ± 2.628	27.538 ± 2.628	12.889 ± 2.039	211.57 ± 1.73	429.41 ± 3.46	11.80	0.001
	2-rate incline-ground 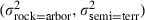	1.498 ± 0.376	10.723 ± 1.689	35.950 ± 3.899	35.950 ± 3.899	10.723 ± 1.689	208.71 ± 2.16	423.70 ± 4.32	6.09	0.023
	1-rate no effect 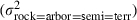	1.384 ± 0.133	19.575 ± 1.043	19.575 ± 1.043	19.575 ± 1.043	19.575 ± 1.043	213.59 ± 1.31	431.31 ± 2.63	13.70	0.001
PC 2	4-rate full 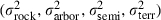	0.244 ± 0.256	0.071 ± 0.122	1.524 ± 0.423	0.265 ± 0.406	0.482 ± 0.190	73.83 ± 1.98	158.37 ± 3.96	2.63	0.074
	3-rate shared tree effect 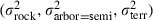	0.209 ± 0.231	0.081 ± 0.136	1.575 ± 0.399	0.450 ± 0.156	0.450 ± 0.156	74.13 ± 2.16	156.73 ± 4.32	0.99	0.168
	3-rate shared ground effect 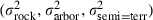	0.204 ± 0.276	0.080 ± 0.145	1.429 ± 0.434	1.429 ± 0.434	0.464 ± 0.204	74.72 ± 2.03	157.91 ± 4.06	2.18	0.093
	3-rate shared incline effect 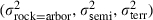	0.145 ± 0.195	0.416 ± 0.150	1.614 ± 0.421	0.324 ± 0.439	0.416 ± 0.150	74.49 ± 2.25	157.45 ± 4.49	1.71	0.117
	2-rate rock effect 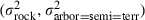	0.503 ± 0.140	0.043 ± 0.064	0.773 ± 0.043	0.773 ± 0.043	0.773 ± 0.043	76.67 ± 1.17	159.61 ± 2.35	3.88	0.040
	2-rate terrestrial effect 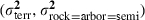	**0.136 ± 0.172**	**0.404 ± 0.122**	**1.632 ± 0.387**	**0.404 ± 0.122**	**0.404 ± 0.122**	**74.73 ± 2.38**	**155.74 ± 4.76**	**0.00**	**0.275**
	2-rate semi-arboreal effect 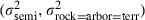	0.397 ± 0.035	0.736 ± 0.037	0.736 ± 0.037	0.051 ± 0.218	0.736 ± 0.037	77.32 ± 1.23	160.92 ± 2.45	5.19	0.021
	2-rate arboreal effect 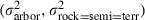	0.163 ± 0.217	1.089 ± 0.349	1.089 ± 0.349	1.089 ± 0.349	0.500 ± 0.205	76.74 ± 1.73	159.75 ± 3.46	4.01	0.037
	2-rate incline-ground 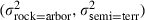	0.110 ± 0.207	0.400 ± 0.156	1.510 ± 0.419	1.510 ± 0.419	0.400 ± 0.156	75.34 ± 2.31	156.97 ± 4.61	1.23	0.149
	1-rate no effect 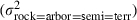	0.375 ± 0.030	0.709 ± 0.032	0.709 ± 0.032	0.709 ± 0.032	0.709 ± 0.032	78.05 ± 1.19	160.24 ± 2.37	4.51	0.029
PC 3	4-rate full 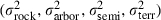	−0.218 ± 0.144	0.032 ± 0.084	1.914 ± 0.475	0.822 ± 0.868	0.382 ± 0.200	70.89 ± 3.77	152.49 ± 7.53	1.12	0.119
	3-rate shared tree effect 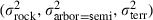	−0.201 ± 0.143	0.034 ± 0.091	1.867 ± 0.414	0.414 ± 0.160	0.414 ± 0.160	71.64 ± 3.53	151.76 ± 7.06	0.39	0.173
	3-rate shared ground effect 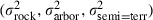	−0.235 ± 0.139	0.030 ± 0.074	1.792 ± 0.430	1.792 ± 0.430	0.370 ± 0.197	71.50 ± 3.85	151.47 ± 7.71	0.11	0.199
	3-rate shared incline effect 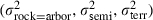	−0.180 ± 0.116	0.337 ± 0.165	1.958 ± 0.477	0.879 ± 0.881	0.337 ± 0.165	71.95 ± 4.21	152.36 ± 8.42	0.99	0.127
	2-rate rock effect 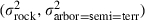	−0.283 ± 0.098	0.036 ± 0.067	0.854 ± 0.044	0.854 ± 0.044	0.854 ± 0.044	77.81 ± 1.15	161.89 ± 2.30	10.52	0.001
	2-rate terrestrial effect 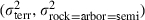	−0.155 ± 0.106	0.373 ± 0.133	1.907 ± 0.420	0.373 ± 0.133	0.373 ± 0.133	72.82 ± 3.91	151.92 ± 7.82	0.55	0.159
	2-rate semi-arboreal effect 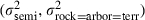	−0.154 ± 0.041	0.799 ± 0.051	0.799 ± 0.051	0.292 ± 0.737	0.799 ± 0.051	79.64 ± 1.34	165.56 ± 2.68	14.19	0.000
	2-rate arboreal effect 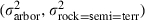	−0.131 ± 0.068	1.382 ± 0.360	1.382 ± 0.360	1.382 ± 0.360	0.400 ± 0.217	75.45 ± 3.37	157.18 ± 6.74	5.81	0.011
	2-rate incline-ground 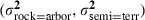	−**0.198 ± 0.113**	**0.327 ± 0.162**	**1.838 ± 0.432**	**1.838 ± 0.432**	**0.327 ± 0.162**	**72.54 ± 4.31**	**151.37 ± 8.63**	**0.00**	**0.210**
	1-rate no effect 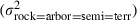	−0.167 ± 0.029	0.777 ± 0.038	0.777 ± 0.038	0.777 ± 0.038	0.777 ± 0.038	80.17 ± 1.08	164.48 ± 2.16	13.12	0.000
PC 4	4-rate full 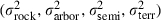	−0.041 ± 0.050	0.007 ± 0.017	0.518 ± 0.056	0.530 ± 0.399	0.236 ± 0.027	52.45 ± 0.79	115.62 ± 1.58	1.86	0.062
	3-rate shared tree effect 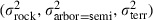	−0.032 ± 0.047	0.006 ± 0.017	0.522 ± 0.054	0.249 ± 0.018	0.249 ± 0.018	52.78 ± 0.76	114.02 ± 1.52	0.26	0.138
	3-rate shared ground effect 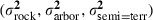	−**0.042**± **0.047**	**0.007**± **0.017**	**0.517**± **0.056**	**0.517**± **0.056**	**0.234**± **0.022**	**52.64**± **0.83**	**113.76**± **1.66**	**0.00**	**0.158**
	3-rate shared incline effect 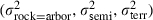	−0.068 ± 0.021	0.209 ± 0.023	0.497 ± 0.053	0.568 ± 0.407	0.209 ± 0.023	53.66 ± 0.76	115.79 ± 1.53	2.03	0.057
	2-rate rock effect 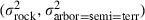	−0.039 ± 0.040	0.010 ± 0.021	0.326 ± 0.016	0.326 ± 0.016	0.326 ± 0.016	53.86 ± 0.62	113.99 ± 1.23	0.23	0.140
	2-rate terrestrial effect 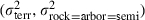	−0.059 ± 0.015	0.223 ± 0.016	0.502 ± 0.054	0.223 ± 0.016	0.223 ± 0.016	54.07 ± 0.71	114.42 ± 1.42	0.66	0.113
	2-rate terrestrial effect 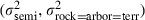	−0.085 ± 0.017	0.286 ± 0.021	0.286 ± 0.021	0.500 ± 0.402	0.286 ± 0.021	55.05 ± 0.51	116.37 ± 1.01	2.61	0.043
	2-rate arboreal effect 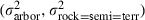	−0.085 ± 0.010	0.384 ± 0.036	0.384 ± 0.036	0.384 ± 0.036	0.237 ± 0.023	54.82 ± 0.61	115.93 ± 1.21	2.17	0.053
	2-rate arboreal effect 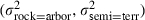	−0.069 ± 0.016	0.208 ± 0.020	0.503 ± 0.057	0.503 ± 0.057	0.208 ± 0.020	53.85 ± 0.81	113.99 ± 1.63	0.23	0.141
	1-rate no effect 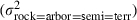	−0.082 ± 0.010	0.293 ± 0.014	0.293 ± 0.014	0.293 ± 0.014	0.293 ± 0.014	55.32 ± 0.48	114.78 ± 0.97	1.02	0.095

PC, principal component; AIC, Akaike information criterion.

Morphological PCs are generally best fit by multiple-rate models that infer evolutionary rates to be slower in rock-dwelling and arboreal lineages than in terrestrial and semi-arboreal lineages. Table 2 presents parameter estimates and fit scores (-ln likelihood and AICc) for each model as means and standard errors taken across 500 habitat reconstructions. Also shown in Table 2 are ΔAICc and Akaike weights (calculated from the mean AICc scores), which served as the basis for choosing the preferred model for each PC. We also compared model fit on each of the 500 habitat reconstructions, and Table 3 reports each model’s mid-95% density interval for ΔAICc as well as the percent of reconstructions for which each model is preferred (ΔAICc = 0.0) and the percent for which each is relatively unsupported (ΔAICc > 2.0).

**Table 3:** Summary of model fit comparisons performed on each habitat and phylogeny reconstruction. Bold denotes best fitting model based on comparison of mean AICc. Note that for any reconstruction only one model is preferred (ΔAICc = 0.0), some models may be disfavoured (ΔAICc > 2.0), and others may receive support (0.0 < ΔAICc < 2.0).

Character	Model	95%ΔAICc interval	% Preferred*	% Disfavoured†
PC 1	4-rate full 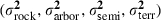	**(0.00, 3.44)**	**77.2**	**9.2**
	3-rate shared tree effect 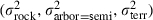	(4.24, 16.79)	0.0	100.0
	3-rate shared ground effect 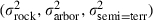	(0.03, 11.76)	2.4	81.6
	3-rate shared incline effect 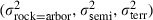	(0.03, 4.75)	2.4	63.8
	2-rate rock effect 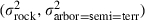	(3.38, 15.47)	0.0	99.8
	2-rate terrestrial effect 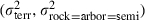	(8.02, 20.72)	0.0	100.0
	2-rate semi-arboreal effect 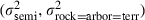	(0.00, 6.28)	17.8	36.6
	2-rate arboreal effect 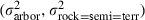	(6.99, 17.91)	0.0	100.0
	2-rate incline-ground 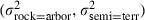	(1.57, 13.43)	0.2	95.6
	1-rate no effect 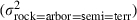	(8.63, 20.71)	0.0	100.0
PC 2	4-rate full 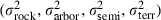	(1.59, 4.26)	0.0	94.2
	3-rate shared tree effect 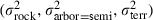	(0.50, 2.64)	0.4	21.2
	3-rate shared ground effect 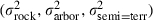	(1.33, 4.83)	0.2	80.0
	3-rate shared incline effect 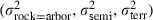	(1.25, 4.34)	0.2	62.4
	2-rate rock effect 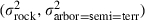	(0.00, 10.57)	20.0	65.4
	2-rate terrestrial effect 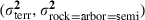	**(0.00, 3.13)**	**72.6**	**14.4**
	2-rate semi-arboreal effect 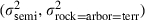	(0.25, 12.38)	2.2	79.4
	2-rate arboreal effect 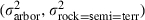	(1.77, 7.37)	0.0	95.4
	2-rate incline-ground 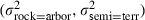	(0.00, 3.58)	3.0	38.2
	1-rate no effec 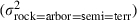	(0.15, 11.25)	1.4	69.8
PC 3	4-rate full 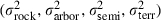	(0.69, 4.23)	0.4	67.4
	3-rate shared tree effect 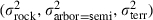	(0.00, 5.08)	18.2	34.8
	3-rate shared ground effect 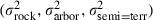	(0.00, 3.97)	3.2	28.2
	3-rate shared incline effect 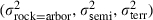	(0.48, 7.06)	0.8	42.2
	2-rate rock effect 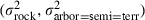	(0.00, 22.15)	15.2	80.8
	2-rate terrestrial effect 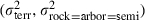	(0.00, 5.90)	14.8	34.4
	2-rate semi-arboreal effect 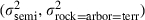	(2.31, 26.79)	0.2	98.4
	2-rate arboreal effect 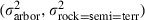	(3.16, 10.18)	0.0	100.0
	2-rate incline-ground 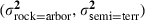	**(0.00, 5.76)**	**47.2**	**27.2**
	1-rate no effect 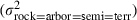	(1.47, 24.89)	0.0	95.8
PC 4	4-rate full 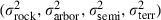	(1.44, 3.38)	0.2	82.2
	3-rate shared tree effect 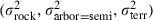	(0.00, 2.04)	10.4	2.6
	3-rate shared ground effect 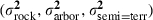	**(0.00, 1.49)**	**25.8**	**1.2**
	3-rate shared incline effect 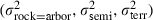	(1.45, 4.12)	0.2	83.2
	2-rate rock effect 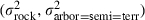	(0.00, 3.04)	32.2	10.8
	2-rate terrestrial effect 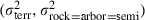	(0.00, 2.64)	4.6	11.2
	2-rate semi-arboreal effect 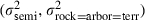	(1.30, 5.56)	0.0	86.4
	2-rate arboreal effect 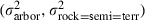	(1.56, 4.72)	0.0	79.2
	2-rate incline-ground 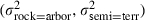	(0.00, 2.39)	21.4	7.0
	1-rate no effect 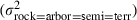	(0.00, 4.04)	5.2	27.6

PC, principal component; AIC, Akaike information criterion.

*Percentage of habitat and phylogeny reconstructions for which ΔAICc = 0.0.

†Percentage of habitat and phylogeny reconstructions for which ΔAICc > 2.0.

In general, models that allow habitat-associated rate variation are more strongly supported than the single-rate model. The best fitting multiple-rate model is much more strongly supported on average than the single-rate model for PCs 1, 2 and 3; for PC 4, several multiple-rate models provide better fit than the single-rate model, but the single-rate model receives substantial support (Table 2). Looking across reconstructions, we found that for all PCs the single-rate model is preferred in 5% or fewer of the reconstructions. In addition, the single-rate model is unsupported in the vast majority of reconstructions for PCs 1, 2 and 3 (100%, 70% and 96%, respectively; also see Fig. S1 for histograms of the single-rate model’s ΔAICc for each PC). The superior fit of the multiple-rate models over the single-rate model supports the hypothesis that rates of morphological evolution vary in association with habitat.

Size evolution in agamids is best fit by the four-rate model, which infers an exceptionally high rate associated with semi-arboreality (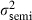 = 146.07 ± 46.02), a slower but intermediate rate for terrestriality (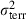 = 20.33 ± 4.67), and yet slower rates for arboreality (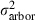 = 13.22 ± 2.15) and rock-dwelling (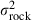 = 2.24 ± 2.06). This model is strongly preferred on average over seven of the other models (ΔAICc > 4; Table 2) and provides the best fit for 77% of the habitat reconstructions (also see Fig. S2 for histograms of ΔAICc for the models preferred for each PC). However, the four-rate model is only somewhat preferred over the two-rate semi-arboreal effect model (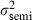 = 166.73 ± 51.75, 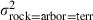 = 14.13 ± 0.86, ΔAICc = 1.42), which provides the best fit for 18% of reconstructions. In addition, there is some support for the three-rate shared incline effect model (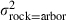 = 11.59 ± 1.96, 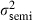 = 148.40 ± 48.17, 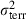 = 20.29 ± 4.65, ΔAICc = 1.94), but this model provides the best fit for only 2% of reconstructions. Each of the three models receiving support infers an elevated rate of evolution in semi-arboreal lineages, and the two best fitting models estimate a separate, intermediate rate for terrestriality.

Evolution of PC 2 is best described by the two-rate terrestrial effect model, which infers a four-fold higher evolutionary rate associated with terrestriality (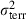 = 1.63 ± 0.39) compared to the rate shared by other habitat types (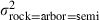 = 0.40 ± 0.12). This model provides the best fit in 73% of habitat reconstructions (also see Fig. S2); however, on average it is only somewhat preferred over the three-rate shared tree effect model (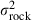 = 0.08 ± 0.14, 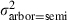 = 0.45 ± 0.16, 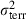 = 1.58 ± 0.40, ΔAICc = 0.99), which is preferred in 20% of the reconstructions. Other models receiving support are the two-rate incline-ground model (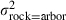 = 0.40 ± 0.16, 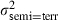 = 1.51 ± 0.42, ΔAICc = 1.23), and the three-rate shared incline effect model (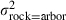 = 0.42 ± 0.15, 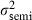 = 0.32 ± 0.44, 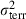 = 1.61 ± 0.42, ΔAICc = 1.71), though these models provide the best fit for only 3% of the reconstructions. Nevertheless, models that receive at least moderate support are similar in that they infer rates of PC 2 evolution to be higher in terrestrial lineages than in rock-dwelling and arboreal lineages.

The two-rate incline-ground model best fits the evolution of PC 3 and estimates a shared evolutionary rate for rock-dwelling and arboreal lineages (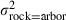 = 0.33 ± 0.16) that is nearly six times slower than the rate shared between semi-arboreal and terrestrial lineages (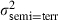 = 1.84 ± 0.43). On average this model is only weakly or moderately supported over the three-rate models (shared tree effect: 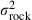 = 0.03 ± 0.09, 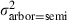 = 0.41 ± 0.16, 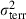 = 1.87 ± 0.41, ΔAICc = 0.39; shared ground effect: 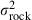 = 0.03 ± 0.07, 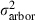 = 0.37 ± 0.20, 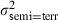 = 1.79 ± 0.43, ΔAICc = 0.11; shared incline effect: 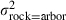 = 0.34 ± 0.17, 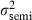 = 0.88 ± 0.88, 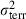 = 1.96 ± 0.48, ΔAICc = 0.99), the two-rate terrestrial effect model (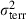 = 1.91 ± 0.42, 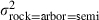 = 0.37 ± 0.13, ΔAICc = 0.55), and the four-rate model (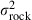 = 0.03 ± 0.08, 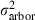 = 0.38 ± 0.20, 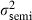 = 0.82 ± 0.87, 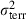 = 1.91 ± 0.48, ΔAICc = 1.12). The two-rate incline-ground model was the most commonly preferred model but is the best fit for only 47% of habitat reconstructions (see Fig. S2). Other models that provide the best fit for a substantial proportion of reconstructions include the three-rate shared tree effect model, the two-rate terrestrial effect model and the two-rate rock effect model, whose fit varied widely among reconstructions (Table 3). Despite the variability in model fit across reconstructions, the models that consistently receive support share similarities in their parameter estimates; the evolutionary rate associated with terrestriality is greater than the rates associated with arboreality and rock-dwelling.

On average, PC 4 is best fit by the three-rate shared ground effect model, which infers the shared evolutionary rate for semi-arboreality and terrestriality (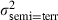 = 0.52 ± 0.06) to be much higher than the rate for arboreality (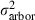 = 0.23 ± 0.02) and rock-dwelling (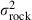 = 0.01 ± 0.01). However, support is quite evenly distributed among the 10 models examined (Tables 2 and 3). In fact, five models receive only somewhat less support than the preferred model, including the three-rate shared tree effect (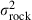 = 0.01 ± 0.01, 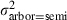 = 0.25 ± 0.02, 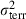 = 0.52 ± 0.05, ΔAICc = 0.26), two-rate rock effect (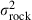 = 0.01 ± 0.01, 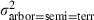 = 0.33 ± 0.02, ΔAICc = 0.23), two-rate terrestrial effect (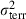 = 0.50 ± 0.05, 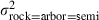 = 0.22 ± 0.02, ΔAICc = 0.66), two-rate incline-ground (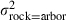 = 0.21 ± 0.02, 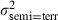 = 0.50 ± 0.06, ΔAICc = 0.23) and single-rate (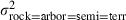 = 0.29 ± 0.01, ΔAICc = 1.02) models. Moreover, although it is preferred on average, the three-rate shared ground effect model is not the most commonly preferred among reconstructions; it is best fit for 26% whereas the two-rate rock effect model is best fit for 32%. Two additional models—the three-rate shared tree effect and two-rate incline-ground models—also provide the best fit to a substantial proportion of reconstructions (Table 3). Although the single-rate model receives at least moderate support for a large proportion of reconstructions, multiple-rate models consistently provide better fit to the evolution of PC 4 (Table 3).

**Fig. 3:**
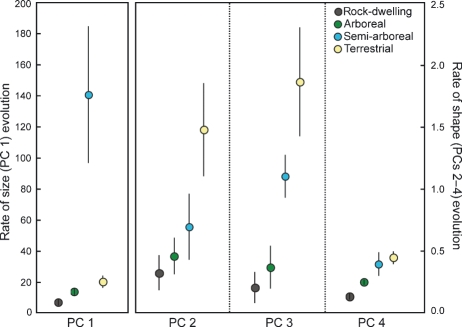
Model-averaged estimates for the rates of PC evolution in rock-dwelling (grey), arboreal (green), semi-arboreal (blue) and terrestrial (yellow) lineages. Point estimates are means of rate estimates from the 10 multiple-rate models and have been weighted by Akaike weights. Error bars are standard errors, representing uncertainty in habitat and phylogenetic reconstructions. Note that the *y*-axis for PC 1, an axis of size variation, is different from the *y*-axis for PCs 2, 3 and 4, which describe shape.

In spite of the ambiguity in selecting the absolute best fitting multiple-rate model, the better fitting models are those that allow rates of evolution to be faster in lineages inferred to be terrestrial or semi-arboreal. This pattern is captured in comparisons of the model-averaged estimates of the rates of PC evolution for each habitat type, which account for uncertainty in model selection. Comparisons of these rate estimates reveal a consistent pattern across PCs that describe limb and body shape variation (PCs 2, 3 and 4); terrestrial lineages have experienced the fastest rates of evolution, semi-arboreality is associated with intermediate evolutionary rates, and arboreal and rock-dwelling lineages evolve at similarly slow rates, though rates are slower for rock-dwelling (Fig. 3). The pattern is somewhat different for model-averaged rates of size (PC 1) evolution; semi-arboreal lineages exhibit a much higher rate than the other three habitat types, though terrestriality is associated with a somewhat higher rate than arboreality and rock-dwelling (Fig. 3).

## Discussion

Habitat use has had strong effects on the evolution of limb and body form in agamid lizards, suggesting that habitats contribute differently to ecological diversification. Models that allow the rate of morphological evolution to vary between lineages that use different habitats provide better fit to the distribution of species’ trait values than the single-rate model, which constrains the rate to be the same in all agamid lineages (Tables 2 and 3). Although we found ambiguity in selection of the preferred multiple-rate model, the better fitting models are generally similar in that they infer slower rates of morphological evolution in rock-dwelling and arboreal lineages than in terrestrial or semi-arboreal lineages (Table 2). Moreover, model-averaged estimates of the rates of evolution for PCs that describe morphological shape (PCs 2–4) reveal a clear and consistent pattern of rate variation associated with habitat states: terrestrial lineages evolve fastest, semi-arboreal lineages evolve at an intermediate rate, and arboreal and rock-dwelling lineages experience similarly slow rates (Fig. 3). For PC 1, which describes variation in size, semi-arboreality is associated with the highest rate, though arboreality and rock-dwelling again exhibit the slowest rates of size evolution (Fig. 3). These results suggest that terrestrial habitats facilitate microhabitat differentiation or evolution along additional ecological axes, whereas the use of trees or rocky surfaces impedes such diversification.

### Diversification within habitat categories

**Fig. 4:**
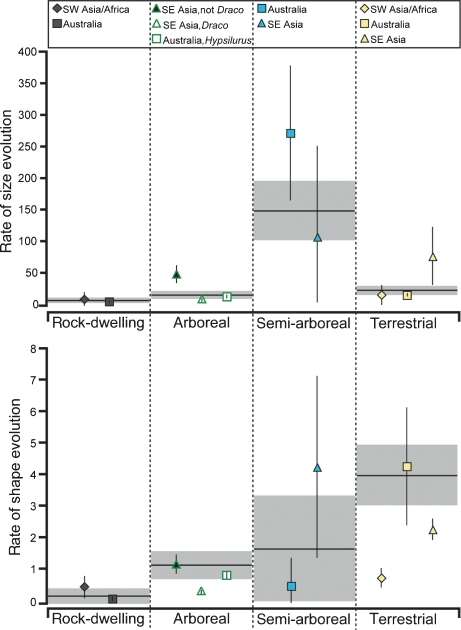
Rates of size and shape evolution for each habitat category estimated within the major agamid clades. Point estimates for the rates of size evolution are means of estimates for the rates of PC 1 evolution across the 500 habitat reconstructions, and estimates for the rates of shape evolution are sums of the mean rate estimates for PCs 2, 3 and 4. Error bars are standard errors for the rate estimates across the 500 reconstructions. Shapes correspond to clade identity (see Fig. 1): diamonds are southwest Asian/African lineages, triangles are southeast Asian lineages, and squares are Australian lineages. Horizontal lines represent rate estimates for all Agamidae (based on the full, four-rate Brownian model), and grey boxes represent ± one standard error taken across reconstructions.

The effects of structural habitat use on diversification occur across multiple lineages that have independently derived these habitat use types. Nearly all stochastic character maps sampled from the posterior distribution infer multiple gains of each habitat use type, and given this set of habitat reconstructions, the best fitting models are those in which rates of morphological evolution vary with habitat (Table 3). Moreover, transitions to each habitat type are inferred to have occurred independently in clades that represent agamid radiations in different geographical regions (see Fig. 1). Multiple transitions across phylogenetically and geographically distant lineages allow the opportunity to evaluate the generality of the effects of habitat use on diversification and to detect the possible influence of lineage-specific effects unrelated to habitat (Read & Nee, 1995). To this end, in the following paragraphs, we review the major groups included in each of the habitat categories and qualitatively compare habitat-associated rate estimates in the major agamid clades (see Fig. 4).

Terrestrial species are spread across three phylogenetically distant agamid groups. The Australian agamid radiation is renowned for its ecomorphological diversity (Pianka, 1986; Melville *et al.*, 2001, 2006; Harmon *et al.*, 2003), and most of the 70 recognized species within the clade are terrestrial, including forms as diverse as *Moloch*, *Pogona* (bearded dragons), the spindly legged *Diporiphora*, *Tympanocryptis* and a variety of *Ctenophorus*. Indeed, the rate of shape evolution is inferred to be higher in the Australian radiation than in the other major continental radiations (Fig. 4). In addition, southeast Asian terrestrial agamids include not only the species rich *Calotes*, but also some highly disparate taxa from the Indian subcontinent and Sri Lanka (e.g. *Otocryptis*, *Sitana* [e.g.Manamendra-Arachchi & Liyanage, 1994]). The terrestrial lineages of the southeast Asian clade exhibit a high rate of size evolution relative to other terrestrial agamid lineages (Fig. 4). The rate of shape evolution in this clade, however, is somewhat lower than the estimate across all terrestrial agamids, though shape evolves more rapidly in terrestrial than in arboreal southeast Asian agamids (Fig. 4). By contrast, the southwest Asian/African group including *Trapelus* and *Pseudotrapelus* contains lesser variation and has experienced substantially lower rates of shape evolution than terrestrial species in the other continental radiations, though the rate of shape evolution in these lineages is somewhat elevated relative to southwest Asian/African rock-dwellers (by about a factor of two; see Fig. 4). We note, however, that *Trapelus* and another terrestrial lineage within this clade, *Phrynocephalus*, are not well sampled in our study. Also, by one account *Pseudotrapelus siniatus* is considered to be rock-dwelling, rather than terrestrial (El Din, 2006), which would lessen our sample for estimating the rate of evolution associated with terrestriality in this clade.

Relatively few semi-arboreal species are included in the data set, none forming large clades. However, many of these taxa are quite distinct, including the two extremely large and semi-aquatic *Physignathus* species, which turn out not to be closely related (Fig. 1; Macey *et al.*, 2000; Hugall *et al.*, 2008). In addition, *Chlamydosaurus* (the frilled lizard) and the long-headed, long-legged *Lophognathus* are closely related, but highly morphologically disparate. The high rate of size evolution in semi-arboreal agamids seems to be largely driven by a high rate in several semi-arboreal Australian lineages, though the elevated rate of shape evolution for semi-arboreality relative to arboreal and rock-dwelling agamids is largely because of a high rate inferred for multiple semi-arboreal southeast Asian lineages (Fig. 4). Within southeast Asia, shape seems to evolve at a faster rate in semi-arboreal lineages than in terrestrial lineages, though the rate associated with semi-arboreality is highly variable across habitat reconstructions. In addition, within Australia, the rate of shape evolution is estimated to be lower in semi-arboreal lineages than in the arboreal clade, *Hypsilurus*, though again there is substantial uncertainty in the rate estimate for semi-arboreal lineages.

Arboreal agamids have generally experienced relatively slow morphological diversification. Low rates are inferred for two large clades, *Draco* and *Hypsilurus* as well as a third paraphyletic group of arboreal species from southeast Asia, including *Gonocephalus*, *Lyriocephalus* and *Ceratophora* (Fig. 4). The estimates for the rates of size and shape evolution in the latter group are somewhat higher than the rates in *Draco* or *Hypsilurus*, but are still lower than the rates in southeast Asian lineages that use other habitat types.

Rock-dwelling agamids have experienced the lowest rates of morphological evolution. In contrast to the high rates in lineages of the Australian radiation that use other habitats, two clades of rock-dwelling *Ctenophorus* species within this larger group have experienced very slow rates of size and shape evolution (Fig. 4). Rock-dwelling lineages of the southwest Asian/African clade are comprised mostly of *Laudakia* species, and the rates of evolution of size and shape in these lineages are nearly as low as in the rock-dwelling *Ctenophorus* lineages (Fig. 4). African rock-dwelling *Agama* are represented by only one species in this study; however, most species in this genus appear morphologically homogeneous, in agreement with trends exhibited by the other rock-dwellers.

For the most part, habitat has consistent effects on diversification in the three major continental radiations of agamids. Although the magnitude of influence of some habitat types varies in the three major continental radiations, these clade and regional effects do not confound our general conclusions about the effects of habitat use. Within the major clades, rates of size and shape evolution are slower in rock-dwelling and arboreal lineages than in lineages that use ground surfaces. Terrestrial lineages of the Australian radiation, which occur primarily in deserts, diversified in shape more rapidly than desert rock-dwellers or forest species. Among southeast Asian taxa—all forest-dwelling—two groups of arboreal species have diversified more slowly than related lineages that occupy other habitat types. Rock-dwelling lineages from southwest Asia/Africa have diversified slowly, but we also note that under-sampling of terrestrial southwest Asian/African species prevents us from ruling out a generally slow rate of morphological evolution in this clade. Finally, we note one exception to the general trend is the somewhat slower rate of shape evolution in semi-arboreal Australian lineages relative to the Australian arboreal clade, *Hypsilurus*. Although rates of size evolution are very rapid in semi-arboreal Australian agamids, rates of shape change in these lineages may be just as slow or even slower than in Australian rock-dwelling and arboreal lineages. However, the rate estimate for semi-arboreality in this clade varies substantially across phylogeny and ancestral reconstructions (see Fig. 4), and thus the rank of this rate estimate relative to arboreal and rock-dwelling lineages is uncertain.

### Mechanisms by which habitat affects diversification

A variety of factors could result in differences in rates of evolution in different structural habitats. We discuss several of these factors in the following paragraphs. We note, however, that at this point we have insufficient data to distinguish among possibilities.

#### Functional constraints

Some structural habitats may impose stricter functional constraints than others, leading to stronger selection resisting diversification away from morphologies that confer effective use of that surface (Simpson, 1953; Butler & King, 2004). In particular, movement and position holding on the steeply inclined surfaces that rocks and trees present to the species that use them may require morphological features and performance abilities that prevent falling (Sinervo & Losos, 1991; Revell *et al.*, 2007; Goodman *et al.*, 2008). In contrast, terrestrial habitats are generally broad and flat, and clinging and climbing are functional considerations that generally apply to a much lesser extent to ground-dwellers. Terrestrial surfaces may therefore impose weaker functional constraints on form and allow for morphology to diverge by neutral evolution or adaptation for other functions (Hansen, 1997; Alfaro *et al.*, 2005). Indeed, ground-dwelling seems to permit a broader variety of forms; an extreme example of this is the thorny devil, *Moloch horridus*, which moves slowly over sandy deserts (Pianka & Vitt, 2003) and possesses short limbs and a wide body that is unique among terrestrial agamid species (Fig. 2a, *M. horridus* has the highest score on PC 2 and the most negative score on PC 3).

#### Habitat complexity

The converse of functional constraints, some structural habitats may provide more ways of making a living. Terrestrial habitats, for example, provide opportunities for burrowers, species that live in leaf litter, in grass, that run quickly in open areas and that remain cryptic against the soil. Terrestrial members of the Australian *Ctenophorus* radiation, for example, have evolved different refuge-seeking strategies, including digging burrows in sand or loose soils or hiding in areas covered by shrubs or grasses (Melville *et al.*, 2001). Burrowing and manoeuvering through structurally complex habitats may impose additional selective demands on locomotor performance that contribute to morphological diversification among terrestrial lineages (Thompson & Withers, 2005). In this way, terrestrial habitats may provide for finer microhabitat differentiation than rocky or arboreal habitats.

In contrast, it is conceivable that fewer ways exist to adapt to rocky or arboreal habitats. Recent work demonstrating convergent and parallel evolution of morphology and performance in rock-dwellers from several different radiations support the hypothesis that there are few ways to make use of rocky habitat (Revell *et al.*, 2007; Goodman *et al.*, 2008). However, ecomorphological diversification in arboreal habitats is well documented in some lizard groups, such as anoles (Williams, 1972; Losos, 2009), geckoes (Pianka & Vitt, 2003; Vitt *et al.*, 2003) and chameleons (Bickel & Losos, 2002), and these groups challenge the generality of our finding that arboreal habitats are diversity limiting. Unlike these groups, agamids lack specialized toe-pads or foot structures, which confer exceptional clinging abilities in the species that possess them (Irschick *et al.*, 1996; Zani, 2000). Relatively limited clinging capabilities may prevent agamids from diversifying to make use of the different structural niches utilized by these other lizard clades.

#### Ecological interactions

If the number of co-occurring, related species is greater in some habitat types than in others, then selection for resource partitioning may lead to adaptive divergence. On the other hand, some habitats may have more competing species of other taxa—such as insectivorous birds or mammals—which may limit the ability of agamids to diversify. Certainly, many terrestrial Australian agamid species occur in sympatry in some areas of the Australian desert, perhaps accounting for diversification in this clade. On the other hand, as many as seven species of the arboreal southeast Asian clade, *Draco*, occur sympatrically (Inger, 1983; McGuire & Dudley, 2005), yet rates of evolution in this clade are low (Fig. 4; though we note that sympatric *Draco* differ in wing size (McGuire, 2003), a morphological attribute which we did not measure). Asian rainforests may have more competing species of other taxa (e.g. birds, small mammals) than Australian deserts—which are known to be dominated by squamates (Pianka, 1986)—and this difference could explain these discrepant patterns.

#### Geographical distribution of species

The converse of large numbers of sympatric species, some habitat types may not facilitate co-occurrence of ecologically similar species. Rather, species may replace each other across the geographical landscape, and thus may occupy the same or similar niches, only in different places. The southwest Asian/African clade is one example in which sympatry of clade members is generally quite low, and the slow rate of morphological evolution in rock-dwelling members of this clade may be a consequence of similar selection pressures acting on members of this clade.

### Caveats

Comparisons of model fit and model-averaged rate estimates provide evidence that morphological diversification varies as a function of habitat use in agamid lineages. We note, however, that the model-fitting approach employed in this study is limited to detecting the best of the evolutionary models we specified to evaluate our hypothesis (Burnham & Anderson, 2002). Therefore, the scope of our conclusions is limited to the relative fit of these models. Although the superior fit of the multiple-rate models over the single-rate models supports the hypothesis of habitat-associated variation in morphological diversification, we cannot exclude the possibility that an alternative, unspecified model provides a better fit to agamid species values and phylogeny. Likewise, our analysis does not rule out roles for other factors that may have influenced morphological evolution in agamid lineages. For example, diversification may also vary with differences in intrinsic factors, such as genetic constraints or origins of novel structures.

A related point concerns the susceptibility of our approach to lineage-specific factors unrelated to habitat that might speed or slow morphological evolution within clades (Read & Nee, 1995). Our conclusions are somewhat protected against such confounding factors because multiple transitions into each of the four habitat use categories are likely to have occurred; however, large clades characterized by a single habitat type (e.g. *Draco*) could exert undue influence on the estimated rate of evolution associated with that habitat. In such a case, we would not be able to disentangle the role of habitat from any other derived factor shared within that clade. Indeed, for this reason we qualitatively assessed possible lineage-specific effects in Fig. 4, but we note that a more general test of habitat’s effects on morphological evolution would involve fitting separate habitat-associated rates within major clades or comparing evolutionary rates between samples of sister clades that differ in habitat use.

In addition, our sampling of agamid species likely influenced the pattern of evolutionary rate variation we document. Our data set represents approximately one-quarter of recognized agamid species diversity, though this proportion is not uniform across the major clades. The under-sampling of species from the southwest Asian/African clade may be the most problematic with respect to our conclusions because at least two primarily terrestrial genera, *Trapelus* and *Phrynocephalus*, may exhibit relatively little morphological disparity. If this is indeed true, their under-representation may have resulted in an inflated estimate for the rate of morphological evolution across terrestrial agamids. However, based on a more extensive morphological data set (J. Schulte, unpublished), disparity in *Trapelus* and *Phrynocephalus* is similar to that of other terrestrial clades that we sampled more extensively in this analysis (e.g. *Tympanocryptis* [size and shape], *Diporiphora* [size]; see Fig. S3). Consequently, rates of evolution in these clades may be comparable to those in other terrestrial clades, and thus their under-representation in our data set may not have biased our results.

## Conclusions

Our results provide compelling evidence that habitat use shapes diversification of limb and body form in Agamidae. The pattern of variation in rates of morphological evolution suggests that terrestrial habitats promote ecological differentiation whereas diversification is slower in rocky and arboreal habitats; however, the precise mechanism by which these habitats contribute differently to diversification remains speculative. We recommend that future research investigate the extent to which functional constraints, habitat complexity or biotic interactions within habitats influence the pattern we document. For example, locomotor performance tests could be applied across a range of agamid forms to determine whether arboreal and rocky surfaces impose more stringent functional demands than terrestrial surfaces. Also, comparisons of the number of co-occurring agamid species within each habitat type could assess whether these habitats present different numbers and types of species interactions. Application of such studies to Agamidae or other animal taxa has the potential to provide further insights into how habitat contributes to differential diversification among evolutionary lineages.

## Supporting information

**Table S1** Agamid species’ GenBank numbers and morphological specimen data.

**Figure S1** Distributions of ΔAICc for the single-rate model for each PC.

**Figure S2** Distributions of ΔAICc for the model that on average best fits each PC.

**Figure S3** Disparity in several groups of primarily terrestrial agamids.

As a service to our authors and readers, this journal provides supporting information supplied by the authors. Such materials are peer-reviewed and may be re-organized for online delivery, but are not copy-edited or typeset. Technical support issues arising from supporting information (other than missing files) should be addressed to the authors.
