# Supplementary material for: Habitat use affects morphological diversification in dragon lizards
Source: J Evol Biol. 2010 May;23(5):1033–49. doi: 10.1111/j.1420-9101.2010.01971.x (PMC2878605; doi:10.1111/j.1420-9101.2010.01971.x)

**Supplementary Table S1** Agamid species’ GenBank numbers and morphological specimen data.

| **Species** | **GenBank No.** | **N_morph_** | **Museum Specimen Nos.** |
| --- | --- | --- | --- |
| *Acanthosaura capra* | AF128498 | 5 | FMNH258715, FMNH258717, FMNH258718, MVZ222129, MVZ222130 |
| *Acanthosaura lepidogaster* | AF128499 | 10 | MVZ224090, MVZ224091, MVZ224092, MVZ224100, MVZ226474, MVZ226476, MVZ226477, MVZ226478, MVZ226479, MVZ236730 |
| *Agama atra* | AF128505 | 11 | CAS175068, CAS193435, CAS193436, CAS196385, CAS199983, CAS200001, CAS200073, CAS201896, MVZ69243, MVZ69244, MVZ75535 |
| *Aphaniotis fusca* | AF128497 | 11 | CAS10946, FMNH176912, FMNH183804, FMNH183805, FMNH183806, FMNH183813, FMNH183819, FMNH183823, FMNH183826, FMNH183827, MNH223209, FMNH81995 |
| *Calotes ceylonensis* | AF128483 | 11 | CAS16878, FMNH108654, FMNH121515, FMNH131409, FMNH131410, FMNH142393, FMNH142394, FMNH167026, FMNH167027, FMNH167028, MCZ32185 |
| *Calotes emma* | AF128487 | 31 | FMNH255492, FMNH255498, FMNH255499, FMNH255504, FMNH255508, FMNH256437, FMNH256438, FMNH258702, GZ35702, GZ35723, GZ35728, GZ35750, GZ35780, GZ35826, GZ35837, MCZ169897, MCZ169898, MCZ169900, MCZ169901, MCZ169902, MCZ169903, MCZ169905, MCZ169906, MCZ169907, MCZ29067, MCZ39064, MCZ39066, MCZ43064, MCZ43065, MCZ43090, MCZ45268, MCZ58841, MCZ7205, MVZ215471, MVZ224102 |
| *Ceratophora aspera* | AF128491 | 5 | MCZ4139, MVZ202470, MVZ202471, MVZ202472, MVZ202473 |
| *Ceratophora stoddarti* | AF128492 | 15 | FMNH120903, FMNH120904, MNH120905, FMNH120906, FMNH131371, FMNH131372, FMNH131373, FMNH131375, FMNH131376, FMNH1585, FMNH178085, MCZ116378, MCZ170496, MCZ39834, MVZ202474 |
| *Ceratophora tennenti* | AF128521 | 7 | FMNH167052, FMNH167053, FMNH167055, FMNH167056, MCZ136187, MCZ4126, MVZ202475 |
| *Chlamydosaurus kingii* | AF128469 | 1 | MVZ81713 |
| *Cophotis ceylonica* | AF128493 | 10 | CAS140754, CAS140755, CAS140756, FMNH124120, FMNH1586, MCZ118595, MCZ25910, MVZ202478, MVZ202479, MVZ202480 |
| *Ctenophorus caudicinctus* | AF375623 | 3 | KU93282, KU93284, R34071 |
| *Ctenophorus decresii* | AF128470 | 1 | R52169 |
| *Ctenophorus femoralis* | AF375627 | 1 | R29313 |
| *Ctenophorus fionni* | AF375638 | 1 | R36628 |
| *Ctenophorus gibba* | AF375625 | 1 | R43604 |
| *Ctenophorus isolepis* | AF375629 | 1 | MVZ81665 |
| *Ctenophorus maculatus* | AF375628 | 1 | R26244 |
| *Ctenophorus maculosus* | AF375621 | 1 | R09415A |
| *Ctenophorus mckenziei* | AF375631 | 1 | R26161 |
| *Ctenophorus ornatus* | AF375624 | 13 | FMNH202557, FMNH202558, FMNH202559, FMNH202560, FMNH202561, FMNH73974, FMNH97076, FMNH97077, FMNH97078, FMNHN12946, FMNHN75417, FMNHN97075, R30081 |
| *Ctenophorus reticulatus* | AF375634 | 1 | R46987 |
| *Ctenophorus rufescens* | AF375636 | 1 | R50171 |
| *Ctenophorus salinarum* | AF375640 | 1 | R43777 |
| *Ctenophorus vadnappa* | AF375639 | 1 | R52321 |
| *Diporiphora australis* | AY133005 | 3 | AMNH86403, MCZ10162, MCZ10538 |
| *Diporiphora bilineata* | AF128473 | 1 | MVZ77646 |
| *Diporiphora lalliae* | AY133007 | 1 | R38771 |
| *Diporiphora linga* | AY133008 | 1 | R32058 |
| *Diporiphora magna* | AY133009 | 1 | AMNH115111 |
| *Diporiphora pindan* | AY133010 | 1 | UMMZ192830 |
| *Diporiphora reginae* | AY133011 | 1 | UMMZ192845 |
| *Draco biaro* | AF288277 | 8 | MCZ170898, MCZ170908, MCZ170909, MCZ170910, MCZ170913, MCZ170914, MCZ170915, MCZ170916 |
| *Draco bimaculatus* | AF288241 | 16 | FMNH105758, FMNH52214, FMNH52217, FMNH52218, FMNH52219, FMNH52220, FMNH52222, FMNH52223, FMNH52225, FMNH52227, FMNH52228, FMNH52229, FMNH52231, FMNH52233, FMNH52235, FMNH63158 |
| *Draco blanfordi* | AF288242 | 16 | CAS221153, CAS221154, FMNH121997, FMNH121998, FMNH121999, FMNH128300, FMNH177087, FMNH177088, FMNH177808, FMNH177809, FMNH177810, FMNH188768, FMNH196078, FMNH228250, FMNH262144, MVZ222155 |
| *Draco boschmai* | AF288269 | 1 | FMNH154836 |
| *Draco bourouniensis* | AF288279 | 1 | CAS136374 |
| *Draco caerulhians* | AF288281 | 14 | MCZ173320, MCZ173321, MCZ173327, MCZ173329, MCZ173330, MCZ173332, MCZ173336, MCZ173337, MCZ173341, MCZ173343, MCZ173344, MCZ173345, MCZ173346, MCZ173347 |
| *Draco cornutus* | AF288244 | 31 | CAS8451, CAS8464, FMNH120033, FMNH120034, FMNH120036, FMNH120039, FMNH129473, FMNH138417, FMNH138422, FMNH150611, FMNH150614, FMNH150620, FMNH158767, FMNH158772, FMNH158777, FMNH158784, FMNH221423, FMNH221424, FMNH221425, FMNH221426, FMNH221427, FMNH221428, FMNH221429, FMNH67330, FMNH67331, FMNH67332, FMNH67333, FMNH67335, FMNH71566, FMNH71567, FMNH71568 |
| *Draco cristatellus* | AF288255 | 4 | FMNH150621, FMNH63701, FMNH63702, FMNH76253 |
| *Draco cyanopterus* | AF288245 | 3 | CAS28200, CAS28339, CAS28349 |
| *Draco fimbriatus* | AF288257 | 22 | FMNH121132, FMNH121133, FMNH143021, FMNH143022, FMNH177230, FMNH178108, FMNH178109, FMNH178110, FMNH178111, FMNH178112, FMNH178114, FMNH184674, FMNH184675, FMNH209575, FMNH209576, FMNH209577, FMNH209578, FMNH209579, FMNH209580, MVZ43521, MVZ43525, MVZ43526 |
| *Draco formosus* | AF288263 | 4 | MCZ16411, MCZ39097, MCZ39098, MCZ390999 |
| *Draco guentheri* | AF288260 | 5 | CAS62499, CAS62500, CAS62501, CAS62502, CAS62503 |
| *Draco haematopogon* | AF288259 | 31 | CAS8460, CAS8461, CAS8462, CAS8463, FMNH11096, FMNH11097, FMNH11098, FMNH11100, FMNH120043, FMNH150627, FMNH150629, FMNH150633, FMNH150637, FMNH150641, FMNH150648, FMNH150655, MNH158701, FMNH158705, FMNH158706, FMNH158707, FMNH158708, FMNH158709, FMNH158710, FMNH158711, FMNH158713, FMNH158714, FMNH158715, FMNH158716, FMNH158721, MVZ111801, MVZ111802 |
| *Draco indochinensis* | AF128477 | 1 | USNM090383 |
| *Draco maculatus* | AF288248 | 9 | FMNH255511, FMNH255589, MVZ226482, MVZ226483, MVZ226485, MVZ23401, MVZ23408, MVZ23414, MVZ236739 |
| *Draco maximus* | AF288231 | 27 | FMNH138414, FMNH138415, FMNH138425, FMNH138428, FMNH145295, FMNH145300, FMNH145305, FMNH150712, FMNH150718, FMNH150721, FMNH150722, FMNH150723, FMNH184676, FMNH184677, FMNH184678, FMNH184680, FMNH221415, FMNH221416, FMNH221417, FMNH221420, FMNH221421, FMNH221422, FMNH240562, FMNH246233, FMNH248964, MCZ39100, MVZ111803 |
| *Draco melanopogon* | AF288258 | 8 | MVZ111804, MVZ111805, MVZ111806, MVZ111807, MVZ111808, MVZ111809, MVZ111810, MVZ111811 |
| *Draco mindanensis* | AF288249 | 8 | CAS133566, CAS133584, CAS24600, CAS24639, FMNH41388, FMNH52243, FMNH63157, FMNH68945 |
| *Draco obscurus* | AF288250 | 29 | FMNH158884, FMNH158886, FMNH158904, FMNH185015, FMNH185016, FMNH185017, FMNH185020, FMNH185022, FMNH185023, FMNH185024, FMNH185029, FMNH185030, FMNH209686, FMNH209687, FMNH209688, FMNH209689, FMNH209690, FMNH209691, FMNH209692, FMNH209693, FMNH209694, FMNH209695, FMNH209696, FMNH209697, FMNH209698, FMNH209699, FMNH209700, FMNH209702, FMNH209703 |
| *Draco ornatus samar* | AF288252 | 2 | USNM533121, USNM533122 |
| *Draco palawanensis* | AF288262 | 5 | CAS157297, CAS157298, CAS28612, CAS28614, CAS28615 |
| *Draco quadrasi* | AF288261 | 15 | CAS185498, CAS64230, FMNH236060, FMNH236061, FMNH236062, FMNH236063, FMNH236064, FMNH236065, FMNH236066, FMNH236067, FMNH236068, FMNH236069, FMNH236070, FMNH251124, FMNH251125 |
| *Draco quinquefasciatus* | AF288232 | 19 | FMNH221322, FMNH221324, FMNH221326, FMNH221329, FMNH221330, FMNH221334, FMNH221343, FMNH221350, FMNH221351, FMNH221354, FMNH221362, FMNH221370, MVZ111816, MVZ111817, MVZ111818, MVZ111819, MVZ111820, MVZ111821, MVZ111822 |
| *Draco reticulatus* | AF288247 | 4 | CAS25099, CAS25101, CAS25102, FMNH202747 |
| *Draco spilopterus* | AF288233 | 1 | USNM513194 |
| *Draco sumatranus* | AF288264 | 2 | USNM129472, USNM129473 |
| *Draco taeniopterus* | AF288251 | 6 | CAS222231, CAS222232, CAS222278, CAS222279, FMNH261744, MVZ111823 |
| *Draco timoriensis* | AF288275 | 1 | FMNH15849 |
| *Draco volans* | AF288267 | 22 | FMNH177337, FMNH177338, FMNH185072, FMNH185075, FMNH185080, FMNH185082, FMNH185084, FMNH185088, FMNH185094, FMNH185095, FMNH185096, FMNH185098, FMNH185102, FMNH185103, FMNH209710, FMNH209711, FMNH209712, FMNH209713, FMNH209714, MVZ111824, MVZ198079, MVZ198081 |
| *Gonocephalus grandis* | AF128496 | 35 | CAS10985, CAS10986, CAS10990, CAS10996, FMNH143146, FMNH171867, FMNH188186, FMNH189034, FMNH206921, FMNH206922, FMNH206923, FMNH206924, FMNH206926, FMNH221591, FMNH221594, FMNH221595, FMNH221596, FMNH221597, FMNH221600, FMNH230165, FMNH230168, FMNH246274, FMNH246275, FMNH246276, FMNH246277, FMNH246278, FMNH246279, FMNH246280, FMNH246281, FMNH246282, FMNH248078, FMNH248079, FMNH248083, MVZ111826, MVZ111829, MVZ111831 |
| *Hypsilurus boydii* | AY133013 | 5 | AMNH27311, FMNH57508, FMNH97320, MCZ111979, MCZ128150, MVZ77648 |
| *Hypsilurus dilophus* | AF128466 | 3 | FMNH14078, MCZ54204, MCZ54205, R11453 |
| *Hypsilurus modestus* | AY133015 | 12 | FMNH14077, MCZ140984, MCZ170247, MCZ170248, MCZ49290, MCZ49291, MCZ49292, MCZ49293, MCZ49294, MCZ49295, MCZ49297, MCZ98700, USNM518560 |
| *Hypsilurus nigrigularis* | AY133016 | 4 | MVZ40785, MVZ40786, MVZ40788, R05561A |
| *Hypsilurus papuensis* | AY133017 | 7 | AMNH92648, MCZ137238, MCZ149685, MCZ153830, MCZ154594, MCZ28660, MCZ44182 |
| *Hypsilurus spinipes* | AY133018 | 1 | FMNH97319, R40742 |
| *Japalura tricarinata* | AF128478 | 13 | CAS177528, CAS177529, CAS177530, CAS177531, CAS177532, CAS177533, CAS177536, CAS177537, CAS177538, CAS177539, CAS177544, FMNH167397, FMNH167398, MCZ58288 |
| *Laudakia caucasia* | AF028683 | 2 | MCZ171961, MCZ97306 |
| *Laudakia nupta* | AF128513 | 3 | MVZ236883, MVZ236884, MVZ236885 |
| *Laudakia sacra* | AF128515 | 3 | MVZ225479, MVZ225481, MVZ225482 |
| *Laudakia stellio* | AF128516 | 3 | MVZ230213, MVZ230214, MVZ97421 |
| *Lophognathus gilberti* | AY133019 | 1 | R42879 |
| *Lyriocephalus scutatus* | AF128494 | 5 | FMNH121213, FMNH166990, FMNH166991, FMNH166992, KU19535, KU19536, MCZ7155 |
| *Moloch horridus* | AF128467 | 4 | MVZ43250, MVZ78828, MVZ81720, MVZ81720, MVZ82832 |
| *Otocryptis wiegmanni* | AF128480 | 9 | CAS16874, CAS16875, CAS16876, CAS16877, FMNH167022, FMNH167023, FMNH167025, FMNH167054, FMNH189017 |
| *Phrynocephalus raddei* | U82691 | 3 | CAS179779, CAS180007, CAS180008 |
| *Physignathus cocincinus* | U82690 | 1 | USNM94610 |
| *Physignathus leusuerii* | AF128463 | 1 | MVZ81712 |
| *Pogona minimus* | AY133022 | 1 | R12786 |
| *Pseudotrapelus sinaitus* | AF128507 | 4 | MVZ198050, MVZ198051, MVZ236932, MVZ97490 |
| *Salea horsefieldii* | AF128490 | 4 | CAS113975, CAS113976, CAS94351, MCZ7198 |
| *Sitana ponticeriana* | AF128481 | 15 | CAS140023, CAS94354, CAS94355, CAS94356, FMNH106802, FMNH106805, FMNH106810, FMNH106811, FMNH106902, FMNH106905, FMNH106906, FMNH171740, FMNH179215, FMNH74944, MCZ7153 |
| *Trapelus agilis* | AF128509 | 6 | MVZ234301, MVZ234303, MVZ236933, MVZ236939, MVZ236940, MVZ236942 |
| *Trapelus sanguinolentus* | AF128511 | 12 | CAS179525, CAS179526, CAS179533, CAS183047, CAS183048, CAS183049, MVZ216014, MVZ216015, MVZ216016, MVZ216017, MVZ216086, MVZ230970 |
| *Tympanocryptis cephalus* | AY133027 | 1 | R42825 |
| *Tympanocryptis intima* | AY133029 | 3 | MCZ51451, MCZ51452, MVZ66562 |
| *Tympanocryptis lineata* | AF128475 | 6 | FMNH73983, FMNH73984, FMNH73985, FMNH97870, FMNH97871, R48306 |
| *Tympanocryptis tetraporophora* | AY133032 | 7 | FMNH256998, FMNH256999, FMNH257000, MCZ182495, MCZ182497, MCZ182498, R48385 |

**Supplementary Figure S1** Distributions of ΔAICc for the single-rate model for each PC. Here, ΔAICc is the difference between AICc for the single-rate model and AICc for the model that best fits each individual habitat reconstruction.


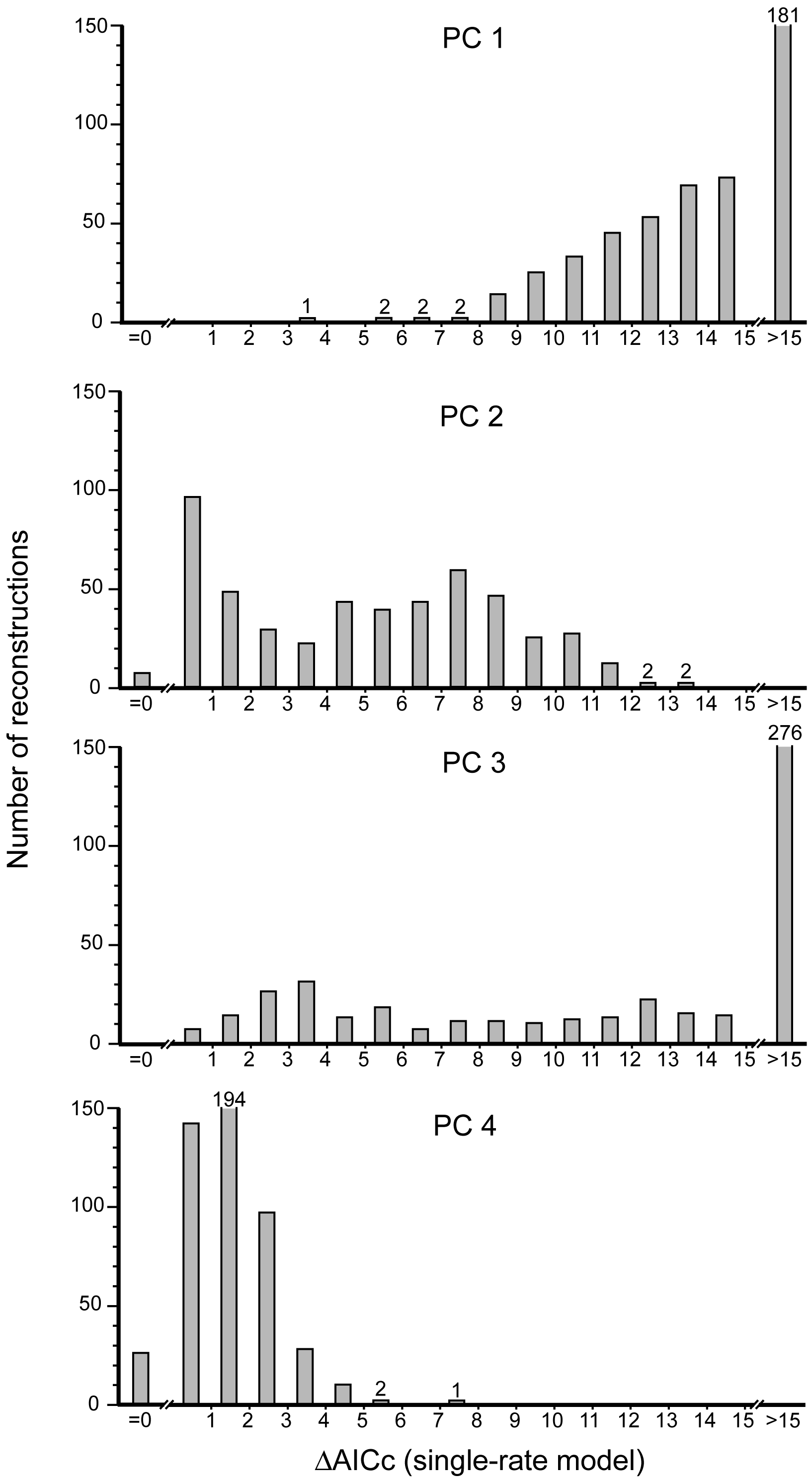


**Supplementary Figure S2** Distributions of ΔAICc for the model that on average best fits each PC. Here, ΔAICc is the difference between AICc for the best model on average and AICc for the model that best fits each individual habitat reconstruction.

**
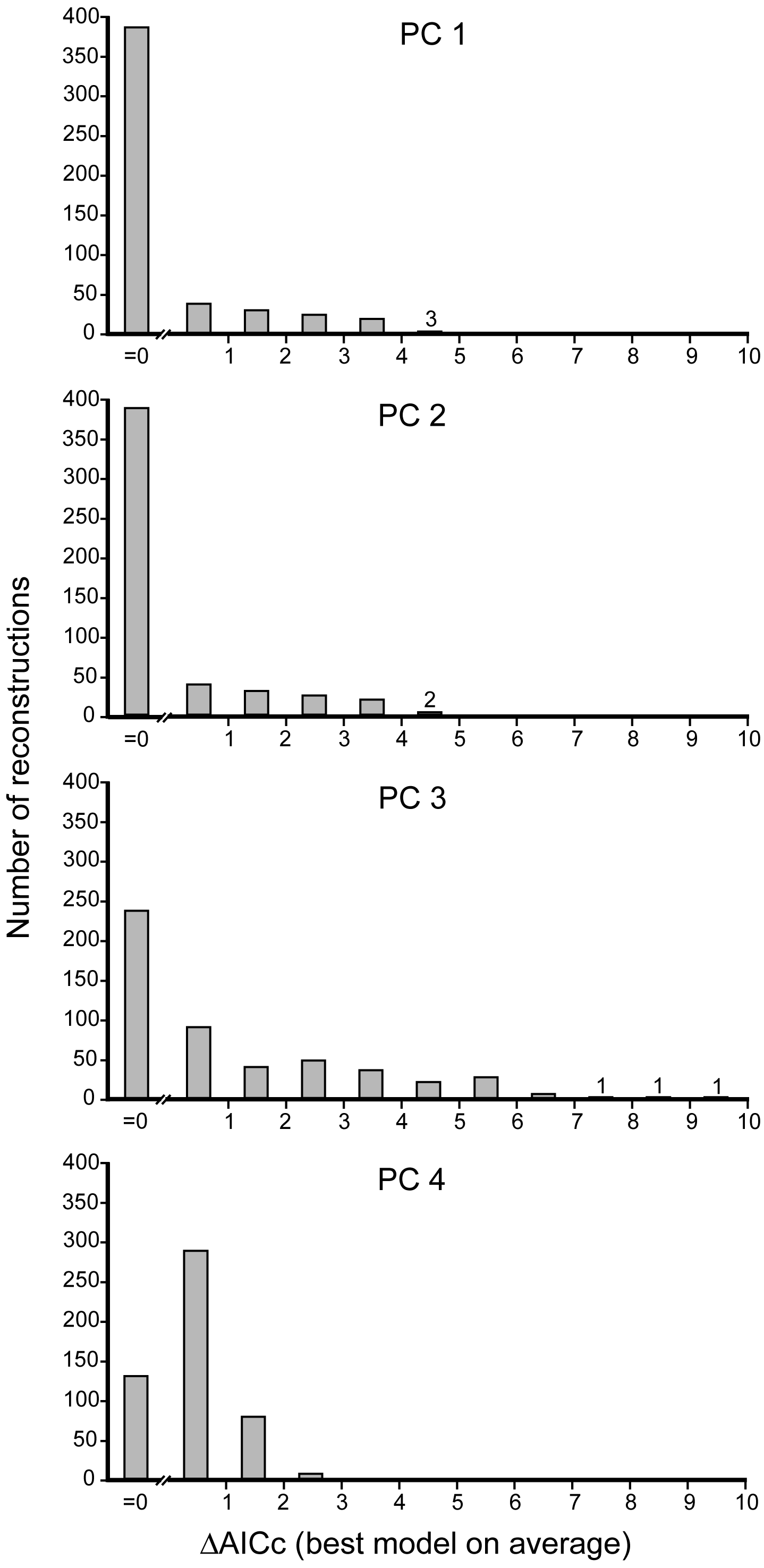
**

**Supplementary Figure S3** Disparity in several groups of primarily terrestrial agamids. Size disparity is reported as within-group variance for PC 1, and shape disparity is the sum of within-group variance for PCs 2, 3, and 4. *Trapelus* and *Phrynocephalus* were under-sampled in our phylogenetic comparative analysis, but we estimated disparity within these groups using a more extensive data set (*n_Trapelus_* = 35, *n_Phrynocephalus_* = 12; J. Schulte, unpublished).


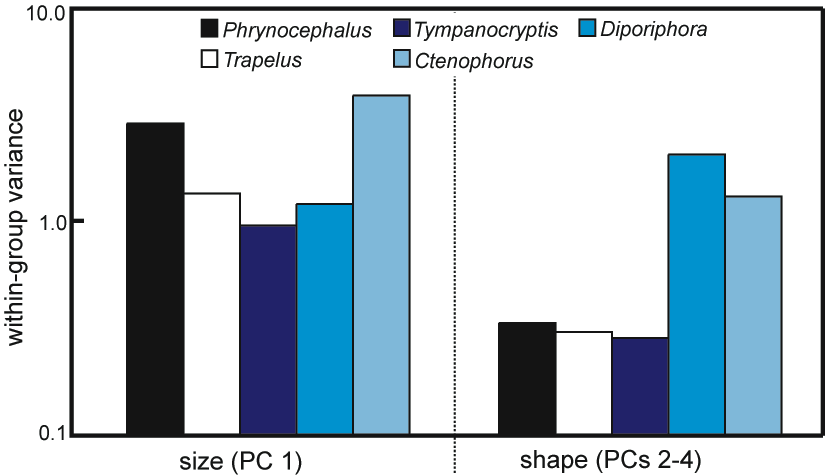

Supplement: Supplementary file 1 [file jeb0023-1033-SD1.doc]
